# Infant and Child Mortality in India in the Last Two Decades: A Geospatial Analysis

**DOI:** 10.1371/journal.pone.0026856

**Published:** 2011-11-02

**Authors:** Abhishek Singh, Praveen Kumar Pathak, Rajesh Kumar Chauhan, William Pan

**Affiliations:** 1 Department of Public Health and Mortality Studies, International Institute for Population Sciences, Mumbai, Maharashtra, India; 2 Department of Geography, Shivaji University, Kolhapur, Maharashtra, India; 3 Population Research Centre, University of Lucknow, Lucknow, Uttar Pradesh, India; 4 Nicholas School of Environment and the Duke Global Health Institute, Duke University, Durham, North Carolina, United States of America; Aga Khan University, Pakistan

## Abstract

**Background:**

Studies examining the intricate interplay between poverty, female literacy, child malnutrition, and child mortality are rare in demographic literature. Given the recent focus on Millennium Development Goals 4 (child survival) and 5 (maternal health), we explored whether the geographic regions that were underprivileged in terms of wealth, female literacy, child nutrition, or safe delivery were also grappling with the elevated risk of child mortality; whether there were any spatial outliers; whether these relationships have undergone any significant change over historical time periods.

**Methodology:**

The present paper attempted to investigate these critical questions using data from household surveys like NFHS 1992–1993, NFHS 1998–1999 and DLHS 2002–2004. For the first time, we employed geo-spatial techniques like Moran's-*I*, univariate LISA, bivariate LISA, spatial error regression, and spatiotemporal regression to address the research problem. For carrying out the geospatial analysis, we classified India into 76 natural regions based on the agro-climatic scheme proposed by Bhat and Zavier (1999) following the Census of India Study and all estimates were generated for each of the geographic regions.

**Result/Conclusions:**

This study brings out the stark intra-state and inter-regional disparities in infant and under-five mortality in India over the past two decades. It further reveals, for the first time, that geographic regions that were underprivileged in child nutrition or wealth or female literacy were also likely to be disadvantaged in terms of infant and child survival irrespective of the state to which they belong. While the role of economic status in explaining child malnutrition and child survival has weakened, the effect of mother's education has actually become stronger over time.

## Introduction

Classical demographic and epidemiological studies examining the level and determinants of infant and child mortality in developing countries have largely focused on the proximate determinants of child survival [1], and ignored the important dimension of geographic space as an independent factor governing the risks of infant and child deaths [2]–[9]. While previous studies have examined the role of child level, maternal level, and household level risk factors of infant and child survival, these studies have largely ignored the potential effect of geographic space in explaining child survival [10], [11]. The limited studies in this area are largely due to inefficiency of the classical survival models that do not appropriately allow for such adjustments. Therefore, by employing a set of geospatial techniques (Moran-*I*, LISA maps and geospatial regression models), and using aggregate level data for 76 natural regions of India over the past two decades, we attempt to examine the potential effect of geographical space on infant and child survival, after adjusting for pertinent socioeconomic and programme-related variables.

India, the most populous country in the South Asian region, contributes to the highest number of deaths among children under-five in the region (2.1 million deaths in 2006) and one-fifth of under-five deaths worldwide [12]. Despite significant progress in infant and child mortality indicators in India in the last two decades [13], [14], current under-five mortality rates are still alarmingly high compared to those of other countries with similar socioeconomic conditions. Moreover, improvements in infant and child morality have been uneven and the brunt of high child deaths is borne by the marginalized and socially disadvantaged section of the population. For example, the Infant Mortality Rate in the poorest 20 percent of the population is 2.5 times higher than that in the richest 20 percent of the population indicating that an infant born in a relatively poor family is more than two times likely to die in infancy than an infant born in a better off family [14]. Not only are the socioeconomic inequalities very high, there are large regional disparities as well; the northern states depicting much higher levels compared to the southern and western states. Even the intra-state disparities are also marked.

Poverty and malnutrition exacerbate the risk of infants and children to various infectious diseases like diarrhea and pneumonia, and heighten the probability of death, particularly among children with low birth weight. Demographic and epidemiological studies have documented that poor economic status of household, low female literacy, poor nutritional status of mother, early age at marriage of mother, large family size, low autonomy of women, and inadequate access to health care services typically lead to disproportionately higher risk for the health status of mothers and their children [15]–[33]. Studies have also documented large socioeconomic and interstate disparities in the maternal and child health status, and have concluded that socioeconomically weaker sections of population disproportionately suffer from poor health status and are also least likely to access health care services [34]–[41]. It is striking to note that the economic inequalities and regional disparities have grown during 1992–2005 [42]–[45].

Regional variations were also marked in the determinants of child survival. The marginal decline in the proportion of population living in poverty in India from 36 percent in 1992–1993 to 27.5 percent in 2004–2005 [46], [47] has been uneven across different states and population subgroups. While the proportion of population living in poverty declined by 24 percent, the percentage of underweight children, on average, declined only by 12 percent [46], [47]. Undernutrition is most pronounced in the states of Bihar, Uttar Pradesh, Madhya Pradesh and Rajasthan with more than half of children being underweight or stunted. Nearly 50 percent of the children in Orissa, Maharashtra and West Bengal are underweight, while 50 percent of the children in Assam and Haryana are stunted [48]. The utilization of safe delivery among mothers in India, on average, increased by 15 percent points (from 26 percent in 1992–1993 to 41 percent in 2002–2004) [49], [50]. However, there exists striking regional divide in the use of safe delivery in India.

A number of demographic studies have highlighted the important role of geographic space in explaining demographic behavior. For example, studies [51] have argued that the conditioning factors of fertility decline in geographically neighboring states in India and Bangladesh, namely, language, identity, historical and cultural similarities transcend political borders and have an independent effect on effecting fertility decline in Greater Bengal (West Bengal in India and Bangladesh). Similar findings were reported in previous studies [52] with reference to the use of fertility control in European countries, where similarities in language along the neighboring states helped to bring about popular use of fertility control methods. Studies [33] have also highlighted the north-south dichotomy in kinship patterns and demographic behavior in India. Evidence presented above does call for models that account for clustering of behaviors over geographic space and time to explain the spatial correlation between outcome and predictor variables over time across contiguous geographic units.

Most of the previous studies have looked at the issue of infant and child mortality using only a broad-set of individual, household level and community level socioeconomic and environmental risk factors ignoring the important effect of geographic space. Such analysis assumes that the relationship between deprivation and mortality is homogenous and uniform over space. However, the findings of studies documenting relationship between deprivation and mortality over space are mixed with some studies documenting homogeneity [53], [54] and others documenting heterogeneity [8], [55]–[58]. So, overlooking the spatial correlations may or may not bias the model results depending on the magnitude of such correlations over time. It is important to note that any aggregation of socioeconomic/demographic/health variables over geographic space tends to exhibit a remarkable spatial pattern/spatial clustering. For example, child undernutrition or child morality portrays a typical geographical distribution/spatial clustering [59]. Ignoring such spatial patterns inflate the uncertainty of the model and makes it less reliable. Fortunately, these limitations can be overcome by the application of geospatial models [60]–[62].

Our literature search could not yield any study that takes into account spatial patterns/clustering while analyzing infant and child mortality in India. To understand this important research gap in demographic and epidemiological literature, we attempt to examine whether the geographic regions which are underprivileged in terms of wealth, female literacy, child nutrition, or safe delivery were also grappling with the elevated risk of child mortality; whether there are any spatial outliers; whether these relationships have undergone any significant change over historical time periods. This study attempts to address these questions using data from large-scale household demographic and health surveys like the National Family Health Survey (NFHS) and the Reproductive and Child Health-District Level Household Survey (DLHS). We use NFHS 1992–1993, NFHS 1998–1999, and DLHS 2002–2004 rounds in the present analysis. The study also attempts to examine the relationship between these variables while adjusting for space and time. We classify India into 76 natural regions based on the agro-climatic scheme proposed by Bhat and Zavier (1999) following the Census of India study [63]. These regions are homogeneous regional clusters in terms of natural topography, agro-climatic conditions and similar cultural attributes.

## Methods

### Ethics statement

The study was based on an anonymous public use data set with no identifiable information on the survey participants; therefore no ethics statement is required for this work.

### Study Design and Data

The data for the present study comes from the first two rounds of the National Family Health Survey (NFHS) conducted during 1992–1993 and 1998–1999 [49], [64] and the second round of the Reproductive and Child Health-District Level Household Survey (DLHS-II, 2002–2004) [50]. These surveys are nationally representative and cover more than 99 percent of the Indian population. The NFHS is an Indian version of the Demographic and Health Survey (DHS) that provides consistent and reliable estimates of fertility, mortality, family planning, utilization of maternal and child health care services and other related indicators at the national, state and regional levels. The DLHS-II is another nationally representative household survey, primarily conducted to monitor and evaluate the implementation of Reproductive and Child Health program across the districts of India. It provides estimates of the coverage of antenatal care and immunization services, safe-motherhood program, family planning program, awareness about RTI/STI and HIV/AIDS, and the utilization of government health services and user's satisfaction, at the district, state and national levels.

The NFHS I (1992–1993) covered 88,562 sample households and 89,777 ever-married women in the 13–49 years age group while NFHS II (1998–1999) covered nearly 91,196 sample households and 89,199 ever-married women in the 15–49 years age group. The overall household response rates improved from 96 percent to 98 percent, while overall individual response rates remained at 96 percent during both rounds of NFHS [49], [64]. The household response rate ranged from 88 percent to 98 percent in NFHS-I, while it varied from 89 percent to 99 percent across the states during NFHS-II. Further, the overall individual response rates ranged from 91 percent to 98 percent across the states during NFHS I, while it varied from 91 percent to 99 percent during NFHS II. On the other hand, DLHS-II (2002–2004) covered around 620107 sample households and 507622 currently married women in the 15–44 years age-group. The overall household response rate (87 percent) ranged from 81 percent to 98 percent while the overall individual response rate (62 percent) varied from 43 percent to 95 percent across the states and union territories in India.

We could not use the third round of NFHS (2005–2006) as it does not provide any opportunity to carry out a regional level analysis mainly due to the unavailability of the district codes in the dataset (which was present in the earlier two rounds) that is crucial to generate geographic regions as per the regional classification proposed by the Census of India [63]. We could not use the third round of DLHS-III (2007–2008) because of the unavailability of information on anthropometric measures of children. Therefore, we used DLHS-II data (very close to NFHS-III time period) that offers a possibility to carry out a regional level analysis owing to its large sample size at the district level and presumably will present a scenario that is similar to NFHS-III. Another advantage of using DLHS-II was that both DLHS and NFHS surveys adopted a similar systematic multistage stratified sampling design, used similar survey instruments (*household and women questionnaires*) which included questions that were phrased alike [49], [50], [64] with similar categories, and both surveys were conducted by the International Institute of Population Sciences (IIPS), Mumbai under the stewardship of the Ministry of Health and Family Welfare (MoHFW), New Delhi. Therefore, owing to these similarities, previous studies have pointed out that it is appropriate to compare the demographic and health estimates generated from these two surveys [65].

### Outcome Variables

We measure two main outcome variables, infant mortality rate (IMR) and under-five mortality rate (U5MR) as these are important indicators of average population health and are widely used to document the progress in the achievement of the fourth Millennium Development Goal (*MDG-4: a commitment to reduce under-five mortality by two-thirds, between 1990 and 2015*). IMR measures the probability of death before the child's first birthday. On the other hand, the U5MR is a measure of the probability of death among children before their fifth birthday. We estimated IMR and U5MR using information on the births and deaths in last ten years preceding each of the survey rounds. We used *ltable* function already available in the STATA to generate IMR and U5MR for each of the 74 natural regions of India over the three survey rounds [74]. We also generated the standard errors for these estimates for all the 74 natural regions over the three survey rounds.

### Exposure Variables

The study utilizes five main exposure variables, that is, poverty, underweight children, female literacy, percent urban and proportion of safe delivery. Measurement of poverty is a complex and debatable issue, particularly due to the unavailability of direct and reliable information on household income or expenditure in sample surveys like NFHS or DLHS. In the absence of such relevant information, studies have successfully used the information on household assets, consumer durables, quality of housing, and access to utilities and infrastructure as proxies to measure the wealth status of the households [74]–[77]. We used the principal component analysis (PCA) to construct wealth quintiles. We then clubbed the lowest two categories of wealth quintiles to create a proxy of poverty status in the respective survey periods (NFHS-I, NFHS-II and DLHS-II). The created proxy was in conformity with the fact that the revised estimates of absolute poverty, that is, head count poverty ratio works out to be 37.3 percent [78] as the weighted average of rural and urban poverty was 41.8 percent and 25.7 percent respectively.

We also estimated the poverty lines using National Sample Survey (NSS) data to examine whether our poverty estimates based on the wealth quintiles differ significantly from the NSS poverty estimates. Consumption based poverty is widely accepted as an absolute and reliable measure of economic welfare at the micro level. The quinquennial rounds of NSS are specifically undertaken to estimate poverty and these samples are representative at the state and sub-state levels separately for rural and urban areas thereof. The state specific poverty cut-off points are defined by the Planning Commission of India taking into account the calories based estimation and price inflation at each five year period. This paper uses data from the sixth and seventh quinquennial surveys undertaken in 1999–2000 and 2004–05 respectively, more commonly known as the 55^th^ and 61^st^ rounds. State specific poverty lines were used to demarcate poor and non-poor households and the proportion of poor households at the agro-climatic regions was estimated.

In order to assess the nutritional status of Indian children below three years of age across 76 natural regions of India in all the three survey rounds, we used the standard anthropometric measure of weight-for-age (Z-score) (underweight, that is, composite index of child nutritional status incorporating stunting and wasting) following the United States-National Centre for Health Statistics (US-NCHS) guidelines [79], [80] due to unavailability of the new WHO reference population [81] in any of the survey rounds included in the analysis. We also note that, on average, the US NCHS standards overestimate the prevalence of underweight children in India by three to five percentage points as compared to the new WHO standards of 2006 [15]. Since the overestimation is likely to happen systematically for every geographic region in each of the survey rounds, the choice of the reference population is not likely to affect our comparisons over the three survey rounds.

We could not use the other two indicators of anthropometric status, that is, height-for-age (stunting) or weight-for-height (wasting) mainly due to two reasons. First, the information on height of children was not collected across several Indian states during NFHS-I, and therefore any all India estimate could be biased due to high non-response rates. Second, the DLHS only provides information on weight-for-age (underweight). Therefore, we have uniformly used the proportion underweight as a measure of child malnutrition across the 76 natural regions in all the three survey rounds. We also estimated the percentage of safe delivery (births delivered in medical facility/attended by medical professional) for each survey round across these 76 natural regions. These estimates are based on the last birth during the three years preceding the survey. Finally, we also measured the proportion of female literates among women in the age group 15–49 years and the proportion urban for each survey round across the 76 natural regions in India.

### Analysis Plan

In order to carry out the regional level analysis, it is important to classify the vast, multifaceted and varied Indian landscape into homogenous groups by applying a standard classification scheme. There are several approaches/schemes for classification of geographic regions based on important characteristics, namely, natural regions based on geographic attributes, agro-climatic and planning regions, socio-cultural and linguistic regions etc [66]–[71]. We adopted the modified scheme of regionalization proposed by Bhat and Zavier (1999) following the Census of India study [63]. Using the district code available in the two rounds of NFHS and DLHS, we classify India into 76 natural regions. The new districts that emerged after the 2001 Indian Census were included and suitably aligned with their parent district(s) after matching the district codes from the 2001 Census publications. The basic rationale behind devising these natural divisions of India was to highlight the inter-regional disparities within states, and similarities between contiguous regions across states, and to demonstrate the role of geographic space as an independent factor that contributes towards the socio-economic, demographic and health status of population, over and above the role of province/state as a modifying factor in bringing desirable changes in population and health parameters. We also note that the sample sizes of these natural regions are sufficient to provide statistically robust estimates on various socioeconomic, demographic and health indicators as demonstrated elsewhere [11], [72]. We use all the 76 geographic regions for descriptive analysis. However, the spatial analysis was restricted only to 74 geographic regions. This is because we could not create estimates for two regions of Jammu & Kashmir (East and West Jammu) in DLHS 2002–2004. In order to maintain uniformity, we restricted our analysis to only those geographic regions for which we could produce estimates of the exposure and outcome variables in each of the three survey rounds.

**Figure 1:**
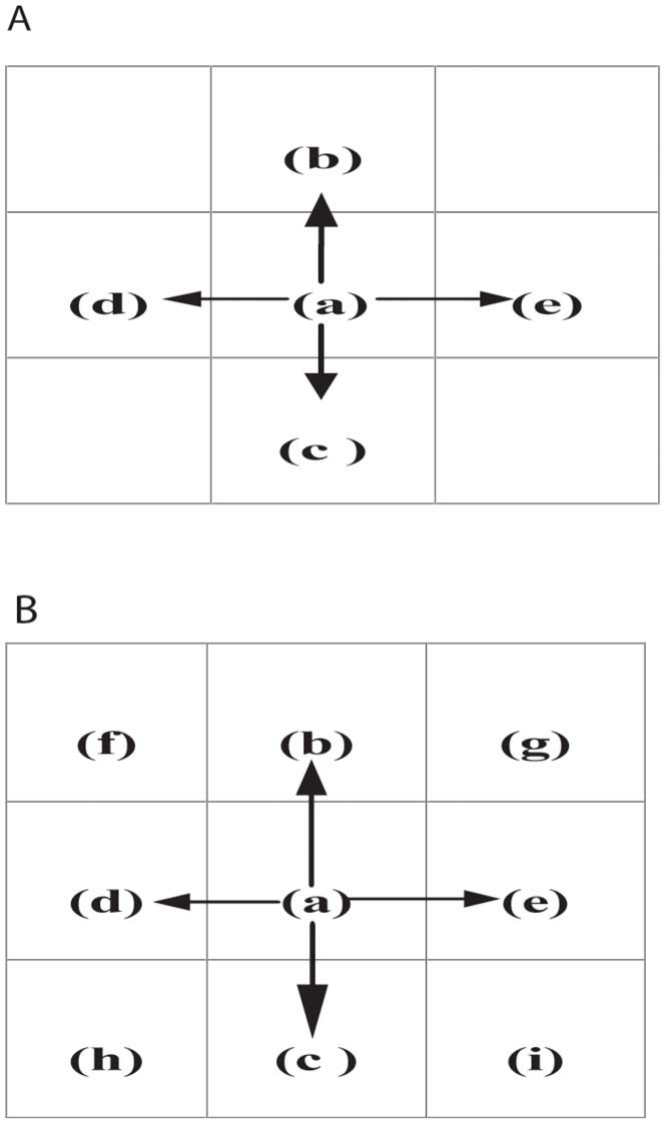
Spatial contiguity weights: Rooks and Queens. **A.** Rook's Weight. **B.** Queens's Weight.

We used the ArcGIS software package to generate the descriptive maps of poverty, child malnutrition, under-five mortality, female literacy, urbanization and safe delivery across 76 natural regions of India over various survey rounds. We then exported the shape files from ArcGIS to GeoDa environment for advanced geospatial analyses. GeoDa is the latest software tool devised by the Centre for Spatially Integrated Social Sciences (CSISS) to implement various exploratory spatial data analysis including data manipulation, mapping, and spatial regression analysis [73]. Using GeoDa software, we generated spatial weights which are essential for the computation of spatial autocorrelation statistics. Essentially, spatial weights can be constructed in two ways: either based on contiguity from polygon boundary files, or based on distance between points. We chose contiguity based spatial weights, since our main interest lies in understanding spatial interdependence between the outcome variable and a set of exposure variables in the neighboring natural regions. GeoDa further provide two types of spatially contiguous weights, that is, rook's weight (uses common boundaries to define neighbor i.e., b, c, d, e) and queen's weight (includes all common points, that is, boundaries and vertices - b, c, d, e, f, g, h, i). We finally used rook's contiguity (**Figure 1**) weight for estimating all the geo-spatial statistics and geo-spatial regressions.

We then employed relevant geo-spatial techniques such as Moran-*I* statistics, Univariate LISA, Bivariate LISA, Geo-spatial regression, and spatio-temporal analysis to address the research questions. The details of the geo-spatial techniques utilized in the paper can be seen in **Appendix S1**.

## Results

### Trends in Outcome and Exposure Variables

**Figure 2:**
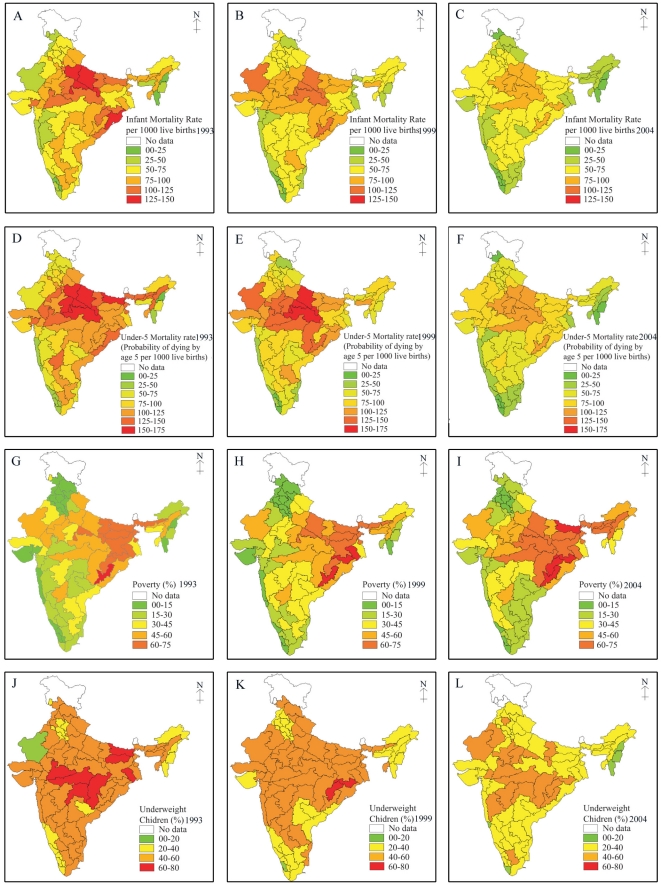
Trends in infant and under-five mortality, poverty and underweight children across 76 geographic regions of India, 1992–2004. **A.** Infant Mortality Rate across 76 regions in India during 1992–1993. **B.** Infant Mortality Rate across 76 regions in India during 1998–1999. **C.** Infant Mortality Rate across 76 regions in India during 2002–2004. **D.** Under-5 Mortality Rate across 76 regions in India during 1992–1993. **E.** Under-5 Mortality Rate across 76 regions in India during 1998–1999. **F.** Under-5 Mortality Rate across 76 regions in India during 2002–2004. **G.** Incidence of Poverty across 76 regions in India during 1992–1993. **H.** Incidence of Poverty across 76 regions in India during 1998–1999. **I.** Incidence of Poverty across 76 regions in India during 2002–2004. **J.** Underweight children (0–35 months) across 76 regions in India during 1992–1993. **K.** Underweight children (0–35 months) across 76 regions in India during 1998–1999. **L.** Underweight children (0–35 months) across 76 regions in India during 2002–2004.

Trends in the dependent and independent variables are presented in **Figure 2**. The trends suggest a sluggish decline in infant and under-five mortality over the last 15 years. IMR declined from 79 per 1000 live births to 58 per 1000 live births in India during 1992–1993 and 2002–2004 respectively, with striking geographical disparities. It varied from 138 per 1000 live births in the South Upland region of Uttar Pradesh to 13 per 1000 live births in Nagaland, with nearly 33 regions recording an IMR well above the national average during 1992–1993. The picture changed gradually over time with decline in IMR. However, regional disparities remained unaffected. For example, IMR varied from 88 per 1000 live births in the Southern Plateau region of Orissa to 12 per 1000 live births in the South Coast region of Kerala during 2002–2004. There were altogether 34 regions where IMR was higher than the national average. On the other hand, U5MR declined from 107 per 1000 live births to 76 per 1000 live births in India during 1992–2004. However, the decline was uneven and largely followed geographical contours. For example, U5MR varied from 197 per 1000 live births in the Southern Upland region of Uttar Pradesh to 16 per 1000 live births in Nagaland in 1992–1993. As in the case of infant mortality, 32 regions stood above the national average in 1992–1993. Even after a fifteen year, the mortality levels remained high along with enormous geographical disparities. Infant and under-five mortality were clustered in the central and eastern regions of India. The clustering has become more pronounced in the central region over the last fifteen years.

In complete contrast to infant and child mortality, the incidence of poverty is found to be clustered in the eastern region and to a relatively lesser extent in the central region. Though the incidence of in India declined during 1992–2004, the regional divide remained stark and wide. In fact, the poverty levels aggravated in some of the eastern and northeastern regions of India. The proportion of underweight children in India also registered a sluggish change over the past fifteen years. Overall, the proportion of underweight children declined from 53 percent to 36 percent in India during 1992–2004 with large regional disparities hidden below the national and state level figures. For instance, the proportion of underweight children varied from 67 percent in Chhattisgarh and Bastar regions of Madhya Pradesh to 15 percent in the South Arid Plain region of Rajasthan. Even in 2002–2004, nearly 18 regions had more than 50 percent of underweight children. On the contrary, we do find significant improvements in female literacy, percent urban, and safe deliveries in almost all the regions of India with a striking spatial gradient during 1992–2004.

### Univariate LISA Results

**Figure 3:**
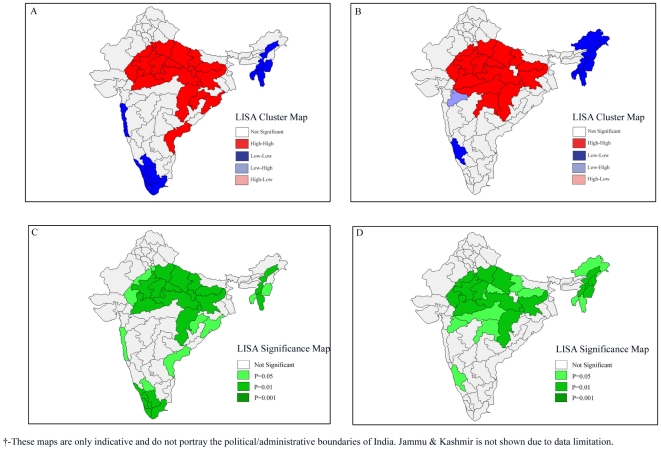
Univariate LISA (Cluster and Significance) maps depicting spatial clustering and spatial outliers of under-five mortality and underweight children across 74 geographic regions of India, 2002–2004. **A.** Univariate LISA Cluster map of under-five mortality rate across 74 regions in India, 2002–2004. **B.** Univariate LISA Cluster map of underweight children across 74 regions in India, 2002–2004. **C.** Univariate LISA Significance map of under-five mortality rate across 74 regions in India, 2002–2004. **D.** Univariate LISA Significance map of underweight children across 74 regions in India, 2002–2004.

The univariate LISA results for under-five mortality and underweight children are presented in **Figure 3**. The LISA cluster map yields four types of geographical clustering of the interest variable. For example, “high-high” means that regions with above average under-five mortality rates also share boundaries with neighboring regions that have above average values of the same variables. On the other hand, “high-low” means that regions with above average under-five mortality rates are surrounded by regions with below average values. The “high-high” are also referred to as *hot spots*, whereas the “low-low” are referred to as *cold spots* [57], [58], [82]. Findings suggest striking geographic clustering of high under-five mortality in the central and parts of eastern India. On the other hand, there were regions with substantially lower under-five mortality rates in some parts of southern and northeastern India. A similar clustering was observed in the case of infant mortality rates (result not shown). We find a clear clustering of underweight children in central, parts of eastern and western India. Unlike clustering of under-five mortality, a majority of regions in northeastern India were characterized by substantially low levels of undernutrition. The central and eastern regions were also characterized by low levels of female literacy, percent urban, and safe deliveries. This trend remained the same in each of the three survey rounds.

### Bivariate LISA Results

**Figure 4:**
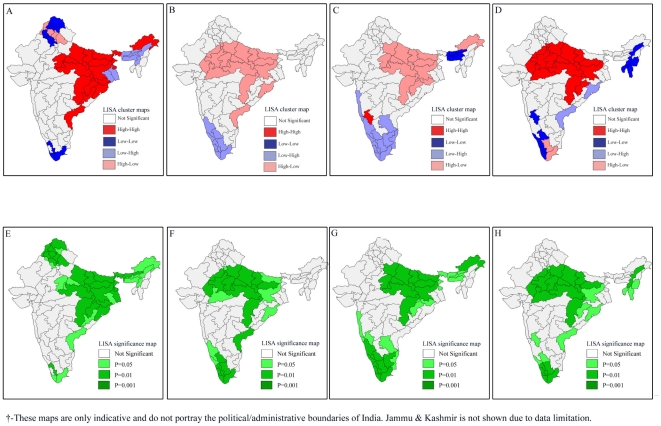
Bivariate LISA (Cluster and Significance) maps depicting spatial clustering and spatial outliers of under-five mortality by poverty, underweight status, female literacy and safe delivery across 74 geographic regions of India, 2002–2004. **A.** Bivariate LISA Cluster map of under-five mortality and poverty across 74 regions of India, 2002–2004. **B.** Bivariate LISA Cluster map of under-five mortality and female literacy across 74 regions of India, 2002–2004. **C.** Bivariate LISA Cluster map of under-five mortality and safe delivery across 74 regions of India, 2002–2004. **D.** Bivariate LISA Cluster map of under-five mortality and underweight children across 74 regions of India, 2002–2004. **E.** Bivariate LISA Significance map of under-five mortality and poverty across 74 regions of India, 2002–2004. **F.** Bivariate LISA Significance map of under-five mortality and female literacy across 74 regions of India, 2002–2004. **G.** Bivariate LISA Significance map of under-five mortality and safe delivery across 74 regions of India, 2002–2004. **H.** Bivariate LISA Significance of under-five mortality and underweight children across 74 regions of India, 2002–2004.

Bivariate LISA examines the spatial relationship between the exposure and the outcome variable for the geographic regions of India. We use bivariate models to address a closely related and pertinent question- whether the geographic regions which were underprivileged in terms of wealth or child nutrition or female literacy or safe delivery were also disadvantaged in terms of infant and child survival. The results presented in **Figure 4** provide compelling evidence that the regions that were poor in economic resources were also more likely to record higher infant and under-five mortality rates. Similarly, we observed some regions where both poverty as well as infant or child mortality were low. Interestingly, we found some regions where we did not get any consistent relationship between access to wealth, and infant and child mortality (**Table S1**). For example, the North-west plateau and South plateau of Karnataka, East Valley and Chachar of Assam and Manipur recorded low incidence of poverty but high levels of infant and under-five mortality. On the other hand, the South Coast of Andhra Pradesh and Delhi registered high incidence of poverty but very low infant and under-five mortality rates.

A similar pattern was observed in the case of child malnutrition and infant and under-five mortality rates. Indeed, the geographic regions that were marked by high child malnutrition levels had strikingly high levels of infant and under-five mortality (**Table S1**). The findings suggest that the geographical regions from central and eastern India were marked by substantially high levels of child malnutrition and infant and under-five mortality rates. Such geographic regions were concentrated in the states of Rajasthan, Madhya Pradesh, Uttar Pradesh, Bihar and Orissa. Surprisingly, there were geographic regions even from these states that did not show the afore-mentioned pattern. In complete contrast to logical reasoning, there were two geographic regions from Tamil Nadu (South East Coast and Kongunad and Nilgiri) which experienced very low levels of child malnutrition but very high levels of infant and under-five mortality. Interestingly, the South Coast of Andhra Pradesh recorded very high levels of child malnutrition but quite low levels of infant and under-five mortality rates.

Bivariate LISA results depicted a similar pattern in the case of female literacy and safe delivery when it comes to explaining infant and under-five mortality. As in the earlier two cases, the geographic regions predominantly from central and eastern India were underprivileged in terms of female literacy or safe delivery, and registered a relatively elevated burden of infant and under-five mortality. The regions predominantly from southern India present a satisfactory pattern with high female literacy and low mortality, and high coverage of safe delivery. However, as in earlier cases, there were outliers in these two cases also.

**Figure 5:**
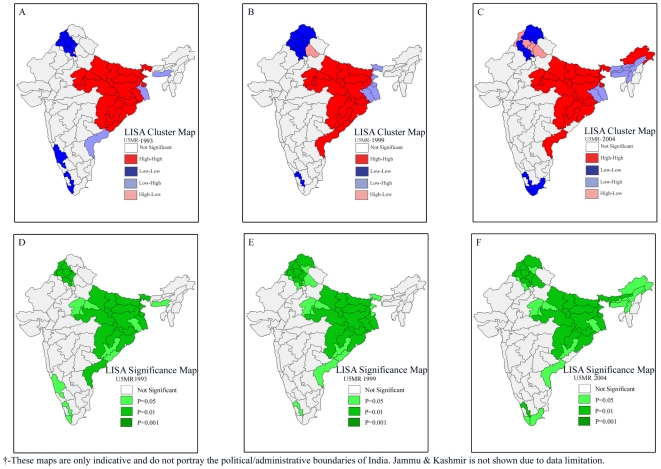
Bivariate LISA (Cluster and Significance) maps depicting spatial clustering and spatial outliers of under-five mortality by incidence of poverty across 74 geographic regions, India, 1992–2004. **A.** Bivariate LISA Cluster map of under-five mortality and poverty across 74 regions of India, 1992–1993. **B.** Bivariate LISA Cluster map of under-five mortality and poverty across 74 regions of India, 1998–1999. **C.** Bivariate LISA Cluster map of under-five mortality and poverty across 74 regions of India, 2002–2004. **D.** Bivariate LISA Significance map of under-five mortality and poverty across 74 regions of India, 1992–1993. **E.** Bivariate LISA Significance map of under-five mortality and poverty across 74 regions of India, 1998–1999. **F.** Bivariate LISA Significance map of under-five mortality and poverty across 74 regions of India, 2002–2004.

**Figure 6:**
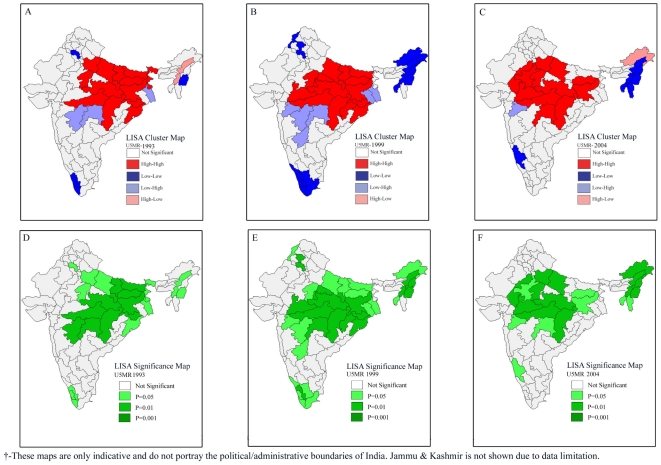
Bivariate LISA (Cluster and Significance) maps depicting spatial clustering and spatial outliers of under-five mortality by prevalence of underweight children across 74 geographic regions, India, 1992–2004. **A.** Bivariate LISA Cluster map of under-five mortality and underweight children across 74 regions of India, 1992–1993. **B.** Bivariate LISA Cluster map of under-five mortality and underweight children across 74 regions of India, 1998–1999. **C.** Bivariate LISA Cluster map of under-five mortality and underweight children across 74 regions of India, 2002–2004. **D.** Bivariate LISA Significance map of under-five mortality and underweight children across 74 regions of India, 1992–1993. **E.** Bivariate LISA Significance map of under-five mortality and underweight children across 74 regions of India, 1998–1999. **F.** Bivariate LISA Significance map of under-five mortality and underweight children across 74 regions of India, 2002–2004.

The trends in the results of bivariate LISA over the three survey rounds suggest a similar pattern in the association of incidence of poverty, female literacy, coverage of safe delivery with infant and under-five mortality (**Figures 5** **,** **6** **,** **7** **,** **8**). However, the afore-mentioned patterns in child malnutrition and infant and under-five mortality did undergo significant changes in some geographic regions over the three survey rounds. It is clear from **Figure 6** that there was an increase in the number of geographic regions recording high child malnutrition and high under-five mortality rates over the three survey rounds. Among geographic regions that show consistent patterns, both hot-spots as well as cold-spots were found. At the same time, we also found geographic regions which clearly show inconsistent patterns.

**Figure 7:**
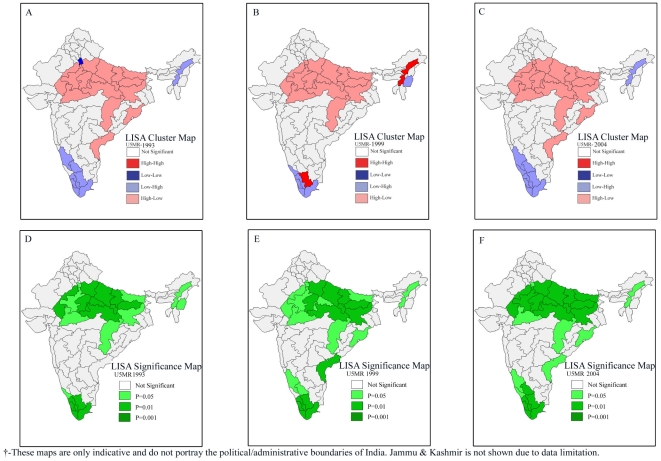
Bivariate LISA (Cluster and Significance) maps depicting spatial clustering and spatial outliers of under-five mortality by female literacy across 74 geographic regions, India, 1992–2004. **A.** Bivariate LISA Cluster map of under-five mortality and female literacy across 74 regions of India, 1992–1993. **B.** Bivariate LISA Cluster map of under-five mortality and female literacy across 74 regions of India, 1998–1999. **C.** Bivariate LISA Cluster map of under-five mortality and female literacy 74 regions of India, 2002–2004. **D.** Bivariate LISA Significance map of under-five mortality and female literacy across 74 regions of India, 1992–1993. **E.** Bivariate LISA Significance map of under-five mortality and female literacy across 74 regions of India, 1998–1999. **F.** Bivariate LISA Significance map of under-five mortality and female literacy across 74 regions of India, 2002–2004.

**Figure 8:**
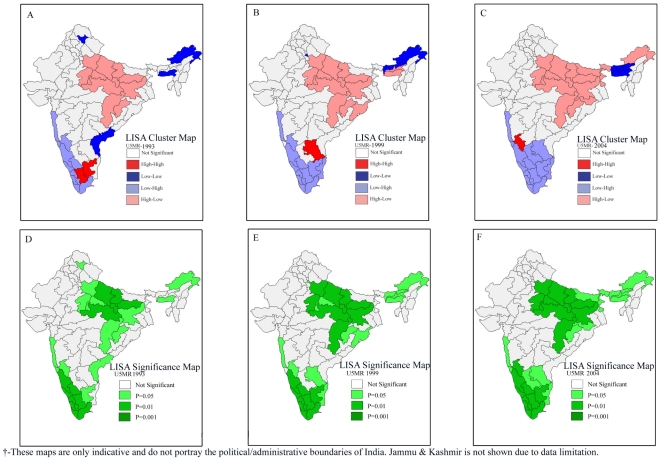
Bivariate LISA (Cluster and Significance) maps depicting spatial clustering and spatial outliers of under-five mortality by safe delivery across 74 geographic regions, India, 1992–2004. **A.** Bivariate LISA Cluster map of under-five mortality and safe delivery across 74 regions of India, 1992–1993. **B.** Bivariate LISA Cluster map of under-five mortality and safe delivery across 74 regions of India, 1998–1999. **C.** Bivariate LISA Cluster map of under-five mortality and safe delivery 74 regions of India, 2002–2004. **D.** Bivariate LISA Significance map of under-five mortality and safe delivery across 74 regions of India, 1992–1993. **E.** Bivariate LISA Significance map of under-five mortality and safe delivery across 74 regions of India, 1998–1999. **F.** Bivariate LISA Significance map of under-five mortality and safe delivery across 74 regions of India, 2002–2004.

### Moran-*I* Results

To account for the magnitude of geo-spatial clustering among the exposure and the outcome variables, we present the Moran-*I* statistics for each of the variables separately for the three survey rounds. The results are presented in **Table 1**. The findings suggest geo-spatial clustering in the exposure and outcome variables in each of the three survey rounds. Infant mortality, under-five mortality, female literacy and safe delivery depicted a substantial amount of geo-spatial clustering in each of the three survey rounds. The proportion of underweight children was not much behind the above-mentioned variables in depicting geo-spatial clustering. The highest geo-spatial clustering was observed in the case of safe deliveries (Moran-*I* = 0.746). In contrast, we did not find any geo-spatial clustering in percent urban over the three survey rounds. A point that must be noted here is that the extent of geo-spatial clustering has followed an increasing trend over the three survey rounds. For example, the Moran-*I* value for infant mortality increased from 0.521 in 1992–1993 to 0.729 in 2002–2004. Similarly, the Moran-*I* values for under-five mortality and safe delivery increased from 0.534 in 1992–1993 to 0.715 in 2002–2004, and from 0.715 in 1992–1993 to 0.746 in 2002–2004, respectively. The Moran-*I* values for child malnutrition show an inconsistent pattern, first depicting an increase in the second round of survey followed by a decrease in the third round.

**Table 1:** Moran-*I* statistics for infant and under five mortality, underweight children, percent urban, female literacy and safe delivery in India, 1992–2004.

Variables	1992–93	1998–99	2002–04
Infant mortality	0.521	0.633	0.729
Under five mortality	0.534	0.662	0.715
Underweight	0.469	0.66	0.485
Percent Urban	0.196	0.234	0.299
Female literacy	0.585	0.554	0.634
Safe delivery	0.715	0.693	0.746

### Ordinary Least Squares (OLS) and Spatial Error Models (Accounting for Space)

**Figure 9:**
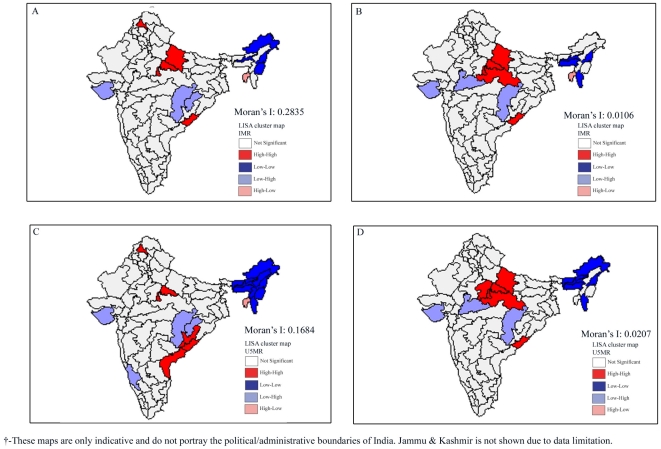
Residual maps of OLS and Spatial Error Models for infant mortality and under-five mortality, India, 2002–2004. **A.** LISA Cluster Map plotting residuals of OLS regression model and Moran's *I* for infant mortality across 74 regions in India, 2002–2004. **B.** LISA Cluster Map plotting residuals of Spatial Error regression model and Moran's *I* for infant mortality across 74 regions in India, 2002–2004. **C.** LISA Cluster Map plotting residuals of OLS regression model and Moran's *I* for under-five mortality across 74 regions in India, 2002–2004. **D.** LISA Cluster Map plotting residuals of Spatial Error regression model and Moran's *I* for under-five infant mortality across 74 regions in India, 2002–2004.

Since we find considerable geo-spatial clustering in the outcome and exposure variables in the three survey rounds, we simultaneously present the results of OLS models and Spatial Error models for infant mortality in **Table 2**. We present the results of Spatial Error models to account for the geo-spatial clustering in the exposure and the outcome variables. Our findings suggest that poverty was significantly and positively associated with infant mortality during the first two survey rounds. However, it lost its significance in the third survey round. Notably, the effect of poverty gradually attenuates over the three survey rounds. In complete contrast to poverty, female literacy depicted an increasingly negative relationship with infant mortality and the association becomes stronger over the three survey rounds. Furthermore, we found an inconsistent relationship between proportion of underweight children and infant mortality. The positive association between percent underweight and infant mortality was significant during 1992–1993 and 2002–2004. However, the relationship was insignificant during 1998–1999. Surprisingly, the coverage of safe delivery and percent urban were not associated with infant mortality in either of OLS or the Spatial Error models. The residual maps presented in **Figure 9** clearly depict the advantage of the Spatial Error models over OLS models. The Moran-*I* value registered an impressive decline from 0.2835 to 0.0106.

**Table 2:** OLS and Spatial Error model to assess the association between infant mortality and socioeconomic variables, India, 1992–2004.

Variable	Aspatial OLS for Infant mortality: 1992–1993		LM Spatial error for Infant mortality: 1992–1993		Aspatial OLS for Infant mortality: 1998–1999		LM Spatial error for Infant mortality: 1998–1999		Aspatial OLS for Infant mortality: 2002–2004		LM Spatial error for Infant mortality: 2002–2004	
	Coefficients	Probability	Coefficients	Probability	Coefficients	Probability	Coefficients	Probability	Coefficients	Probability	Coefficients	Probability
Poverty	0.445	0.004	0.417	0.010	0.313	0.011	0.388	0.006	−0.104	0.148	0.018	0.852
Underweight	0.758	0.004	0.818	0.001	0.219	0.312	0.091	0.683	0.526	0.000	0.261	0.059
Urbanization	0.143	0.457	0.037	0.834	0.169	0.242	0.165	0.174	0.049	0.592	0.063	0.417
Female literacy	−0.533	0.014	−0.476	0.032	−0.535	0.000	−0.375	0.014	−0.699	0.000	−0.594	0.000
Safe delivery	0.003	0.985	0.062	0.738	−0.170	0.109	−0.179	0.147	−0.081	0.288	−0.055	0.528
Constant	39.774	0.008	36.399	0.013	71.147	0.000	68.184	0.000	80.585	0.000	76.975	0.000
Number of observations	74		74		74		74		74		74	
Log likelihood	−323.662		−320.581		−303.338		−296.432		−271.002		−263.000	
AIC	659.323		653.162		618.676		604.863		554.005		538.000	
R square	0.625		0.668		0.680		0.755		0.801		0.855	
Lag Coefficient (Lambda)			0.385	0.000			0.542				0.600	0.000
Breusch-Pagan test for DIAGNOSTICS OF HETEROSKEDASTICITY	3.645	0.601	3.395	0.639	3.645	0.601	9.038	0.108	1.180	0.947	1.266	0.938
Likelihood ratio test			6.161	0.013			13.813	0.000			16.005	0.000

The results of OLS and Spatial Error models for under-five mortality are presented in **Table 3**. Findings reveal diminishing effect of poverty on under-five mortality over the three survey rounds. As in the case of infant mortality, the effect of poverty becomes insignificant in the last survey round. Again, as in the case of infant mortality, female literacy showed an increasingly negative effect over the three survey rounds. Percent underweight children depicted a pattern similar to that observed in the case of infant mortality. The regression diagnostics presented in **Table 3** and the lower panel of **Figure 9** clearly suggest that Spatial Error models are better than OLS models (The Moran-*I* declines from 0.1684 to 0.0207).

**Table 3:** OLS and Spatial Error model to assess the association between under-five mortality and socioeconomic variables, India, 1992–2004.

Variable	Aspatial OLS for Under 5 mortality: 1992–1993		LM Spatial error for Under 5 mortality: 1992–1993		Aspatial OLS for Under 5 mortality: 1998–1999		LM Spatial error for Under 5 mortality: 1998–1999		Aspatial OLS for Under 5 mortality: 2002–2004		LM Spatial error for Under 5 mortality: 2002–2004	
	Coefficients	Probability	Coefficients	Probability	Coefficients	Probability	Coefficients	Probability	Coefficients	Probability	Coefficients	Probability
Poverty	0.623	0.003	0.602	0.007	0.414	0.021	0.435	0.036	−0.031	0.722	0.078	0.485
Underweight	1.052	0.004	1.219	0.000	0.430	0.175	0.238	0.454	0.605	0.001	0.413	0.021
Urbanization	0.108	0.679	−0.024	0.917	0.290	0.169	0.263	0.126	0.109	0.328	0.129	0.213
Female literacy	−0.734	0.012	−0.667	0.025	−0.696	0.001	−0.520	0.018	−0.894	0.000	−0.807	0.000
Safe delivery	−0.028	0.900	0.114	0.654	−0.345	0.027	−0.339	0.056	−0.212	0.025	−0.186	0.085
Constant	55.003	0.006	43.665	0.027	91.596	0.000	92.264	0.000	104.580	0.000	99.950	0.000
Number of observations	74		74		74		74		74		74	
Log likelihood	−346.720		−341.842		−332.004		659.028		−286.593		579.062	
AIC	705.440		695.685		676.992		673.012		585.185		593.046	
R square	0.653		0.712		0.686		0.773		0.843		0.863	
Log Coefficient (Lambda)			0.458	0.000			0.576	0.000			0.432	0.000
Breusch-Pagan test for DIAGNOSTICS OF HETEROSKEDASTICITY	5.327	0.377	4.591	0.468	15.322	0.009	10.188	0.070	3.142	0.678	2.832	0.726
Likelihood ratio test			9.755	0.002			16.970	0.000			6.123	0.013

### Accounting for both Space and Time

We used a longitudinal model that simultaneously adjusts for correlation over space and time. The results of the longitudinal models are presented in **Tables 4** and **Table 5**. The baseline and interaction models both indicate that the percentage of persons poor and underweight and percentage of literate females were significantly related to both the infant mortality and under-five mortality rates. The percent urban and coverage of safe deliveries were not significantly related to either outcome. The baseline model indicates that one percentage unit increase in the percent poor corresponds to a 0.40 and 0.55 unit increase in the IMR and U5MR, respectively; a one percentage unit increase in underweight children corresponds to an increase of 0.60 and 0.82 in the IMR and U5MR; and a one percentage unit increase in literate females corresponds to a decline of 0.25 and 0.36 unit in IMR and U5MR (**Table 4**). Both the IMR and U5MR models demonstrate declining rates over time, with IMR declining from an estimated 66.7 to 62.4 and further to 57.3 during 1992–1993, 1998–1999 and 2002–2004 respectively, and U5MR from 91.6, 85.7, and 76.2 during 1992–1993, 1998–1999 and 2002–2004 respectively.

**Table 4:** Longitudinal models to assess the association between infant and under-five mortality and socioeconomic variables, India 1992–2004.

Covariate & category	Infant mortality	Under-five mortality
Year	9.40**	15.32**
1992–93	5.12	9.49**
1998–99		
2000–04 ®		
Percent poor	0.40***	0.55**
Percent underweight	0.60***	0.82***
Percent urban	0.10	0.14
Percent females literate	−0.25*	−0.36*
Percent safe delivery	−0.02	−0.09

***p<0.001,

**p<0.01,

*p<0.05, (R): Reference category.

**Table 5:** Longitudinal models with interaction terms to assess the association between infant and under-five mortality and socioeconomic variables, India 1992–2004.

Covariate and category	Infant mortality			Under-five mortality		
	Year	Estimate	Change in infant mortality because of a unit change in covariate	Year	Estimate	Change in under-five mortality because of a unit change in covariate
Year	1992–1993	−40.88**		1992–1993	−50.42*	
	1998–1999	2.86		1998–1999	−1.54	
	2002–2004	®		2002–2004	®	
Percent poor		0.16			0.24	
Percent poor*Year	1992–1993	0.44***	0.6107	1992–1993	0.55***	0.7925
Percent poor*Year	1998–1999	0.30**	0.4686	1998–1999	0.31*	0.5510
Percent poor*Year	2002–2004	®	0.1655	2002–2004	®	0.2387
Percent underweight		0.35			0.36	
Percent underweight*Year	1992–1993	0.46	0.8078	1992–1993	0.71*	1.0652
Percent underweight*Year	1998–1999	−0.28	0.0652	1998–1999	−0.05	0.3075
Percent underweight*Year	2002–2004	®	0.3473	2002–2004	®	0.3575
Percent urban		0.09			0.13	
Percent females literate		−0.41**			−0.59**	
Percent females literate*Year	1992–1993	0.31*	−0.1014	1992–1993	0.36	−0.2309
Percent females literate*Year	1998–1999	0.06	−0.3525	1998–1999	0.05	−0.5335
Percent females literate*Year	2002–2004	®	−0.4153	2002–2004	®	−0.5868
Percent safe delivery		−0.08			−0.15	

***p<0.001,

**p<0.01,

*p<0.05, (R): Reference category.

The predicted values, however, change when adjusted for interactions to: 65.6, 62.5 and 57.4 for IMR and 90.9, 86.4, and 76.3 for U5MR. While there was still a declining trend predicted, the decline from 1993 to 1999 is no longer significant for either rate (p = 0.20 for IMR and p = 0.18 for U5MR). The interaction terms are interpreted as the different slopes for the respective relationships each year. For example, the baseline IMR model indicates that a 10-unit increase in the proportion of poverty is associated with a 4.1 unit increase in IMR, but the interaction model indicates that the same 10-unit increase was associated with a 6.1 unit increase in IMR for 1992–1993, a 4.7 unit increase in 1998–1999 and a 1.7 unit increase in 2002–2004 indicating the declining importance for the percent poor influencing IMR (**Table 5**). The same trend is seen for poverty with U5MR; however, this declining trend is not so obvious for percent underweight and female literate. For IMR, the impact of percent underweight children is high in 1992–1993 (8.1 for a 10-unit increase), it declines rapidly to near zero in 1998–1999 (0.7 for a 10-unit increase) and increases again in 2002–2004 (3.5 for a 10-unit increase). For U5MR, the relationship with underweight children also declines rapidly from 1992–1993 to 1998–1999 (10.7 to 3.1 increase in U5MR for a 10-unit increase in underweight), but increases slightly in 2002–2004 (3.6 for a 10-unit increase). Female literacy had a consistently negative relationship with mortality rates that became increasingly important over time, for example, a 10-unit increase in female literacy is associated with a 1.0 unit decline in IMR in 1992–1993, a 3.5 unit decline in 1998–1999, and a 4.2 unit decline in 2002–2004 with the equivalent declines being 2.3, 5.3, and 5.9 for U5MR respectively for the corresponding years. The baseline model indicated a good fit to the data and registered improvement when interactions were considered.

**Figure 10:**
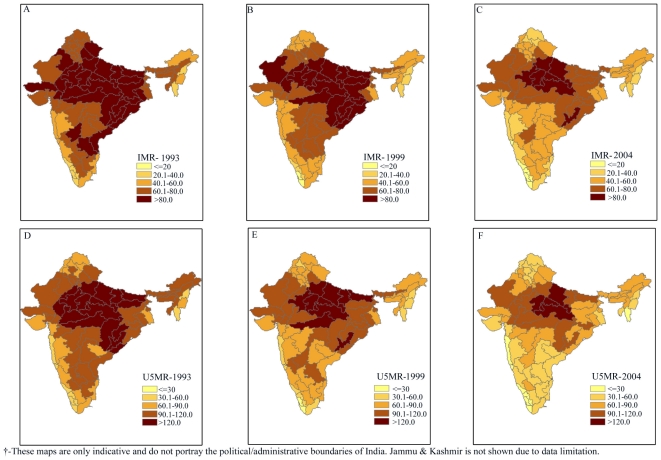
Predicted maps for infant and under-five mortality after accounting for space and time, India 1992–2004. **A.** Predicted Infant Mortality Rate across 74 regions of India, 1992–1993. **B.** Predicted Infant Mortality Rate across 74 regions of India, 1998–1999. **C.** Predicted Infant Mortality Rate across 74 regions of India, 2002–2004. **D.** Predicted Under-five Mortality Rate across 74 regions of India, 1992–1993. **E.** Predicted Under-five Mortality Rate across 74 regions of India, 1998–1999. **F.** Predicted Under-five Mortality Rate across 74 regions of India, 2002–2004.

The result in **Figure 10** presents the predicted maps (after accounting for both space and time) of infant and under-five mortality for the 74 geographic regions of India for each of the three survey rounds. Findings suggest considerable changes in the geographical clustering of infant and under-five mortality rates over the three survey rounds. For example, infant mortality was very high and particularly clustered in the central, eastern, some parts of western and the southern regions during 1992–1993. However, during 2002–2004, very high infant mortality rates were found only in certain geographic regions from central India and one geographic region in eastern India. Almost every region of central and eastern India had relatively higher infant mortality rates compared to other geographic regions of India. The case was similar with under-five mortality. Again, high to very high pockets of under-five mortality were found in central India, some parts of eastern India, and few pockets of western India.

## Discussion

This study is the first investigation that simultaneously accounts for space and time while examining the complex interplay of poverty, child malnutrition, urbanization, female literacy, and safe delivery with infant and under-five mortality in India. This is also a first attempt to address the research question whether the geographical regions which were disadvantaged in terms of poverty, child nutrition, urbanization, female literacy, and coverage of safe delivery were particularly underprivileged in terms of infant and under-five survival. Another novelty of this study is that the unit of analysis is natural geographic region which is a lower level unit compared to the state/province. By doing so, we go below the state level averages that often mask the crux of the problem and thus we highlight the intra-state geographical disparities in infant and under-five mortality. Findings clearly suggest that the regions that were underprivileged in terms of female literacy or child nutrition were also disadvantaged in terms of the two indicators of mortality during early childhood. Moreover, the effect of poverty on infant and under-five mortality attenuated with time, whereas, female literacy had a consistently accentuating effect. Urbanization and coverage of safe delivery were not associated with either infant or under-five mortality. The findings highlight considerable geospatial clustering in the outcome and exposure variables. In addition, the AIC values declined significantly as we moved from ordinary least square regression models to spatial error regression models, thus highlighting the potential advantage of geo-spatial models over classical models.

The National Health Policy (2002) and the National Population Policy (2000) have envisaged at reducing infant mortality to 30 per 1000 by the year 2010 [83], [84]. Our findings suggest that though there has been a decline from 67 per 1000 in 1992–1993 to 57 per 1000 in 2002–2004, the decline has been far from the goal that needs to be achieved. Not only is the decline slow, but there were disparities in such declines across different geographic regions of India. Some geographic regions have achieved very low levels of mortality, whereas other regions still have substantially high levels. The major concern is the clustering of high mortality in certain pockets of the country. Data suggests that India has not succeeded in achieving equitable levels of mortality decline across the country.

A key component of the analysis was to generate poverty estimates over the three survey rounds. We created poverty estimates using two different methodologies. First, we estimated wealth quintiles using the two rounds of NFHS and the second round of DLHS, and then clubbed the lowest two categories together and coded them as poor. Second, we created poverty estimates using NSS data from the 55^th^ (1999–2000) and 61^st^ (2004–05) rounds using the procedure adopted by the Government of India to produce poverty estimates. We intended to use the NSS poverty estimates in the present analysis, but could not do so because of the unavailability of district codes in the 50^th^ round of NSS data collection (conducted in 1993–94) which was the closest round of NSS to our first survey round (1992–1993). Because of the unavailability of district codes, it was not possible for us to construct poverty estimates for the different regions surrounding our first survey round (NFHS 1992–1993). So, in order to maintain uniformity, we used the poverty estimates based on data from NFHS and DLHS. However, we used the poverty estimates created using NSS to check the consistency and reliability of the poverty estimates obtained from the NFHS and DLHS datasets. In addition, we used the poverty estimates created from NSS datasets to examine the association between poverty and infant and under-five mortality after adjusting for child malnutrition, urbanization, female literacy and safe delivery using NFHS 1998–1999 and DLHS 2002–2004. The results obtained using NSS poverty estimates were similar to those obtained from using poverty estimates generated using NFHS and DLHS datasets. The similarity in results reaffirmed our faith in the findings of this study.

A key contribution of the study is the identification of hotspots (that is, regions with high poverty and high infant and under-five mortality; high child malnutrition and high infant and under-five mortality) and cold-spots (regions with low poverty and low infant and under-five mortality; high female literacy and low infant and under-five mortality). There is a general understanding among researchers and policy-makers that infant mortality and under-five mortality are lower in one group of states and higher in another group of states. But this analysis shows that there are huge disparities even within the states. This study clearly brings out spatial contours where the poverty and infant and under-five mortality are clustered, and where child malnutrition and infant and under-five mortality are clustered. The findings depict geographic regions that need immediate and careful attention of the policy makers if India needs to achieve MDG Goal 4. This study also brings out for the first time the fact that geographic regions that were underprivileged in child nutrition were also likely to be disadvantaged in terms of infant and under-five mortality irrespective of the state to which they belong.

Another key finding of the study is the identification of geographic regions that present inconsistent relationships between the exposure variables and outcome variables (that is, regions that showed high poverty but low mortality; regions that showed high malnutrition but lower mortality). For example, the South Coast of Andhra Pradesh showed a typical pattern with high poverty and low mortality; high child malnutrition and low mortality; and high percentage of safe delivery but high mortality. The North West Plateau and South Plateau of Karnataka showed low poverty but high infant and under-five mortality. Similarly, the ‘South East Coast’ and ‘Kongunad and Nilgiri’ of Tamil Nadu depicted low child malnutrition but high mortality during infancy and early childhood. These regions particulary warrant carefully designed studies to understand factors explaining such irregular patterns. Such focus was lacking in the earlier studies conducted either in India or in the other parts of the world.

Another finding of the study that needs attention is the inconsistent relationship between the prevalence of child malnutrition and mortality during infancy and early childhood. The prevalence of child malnutrition was significantly and positively associated with mortality during the first and the last survey rounds, but was insignificant in the second survey round. Results from earlier studies also support our findings [85]. This could happen because there is no one to one correspondence between child malnutrition and mortality. There is a possibility of time-lag between the occurrence of child malnutrition and mortality during infancy and early childhood. This could also be possible because of the existence of mortality selection as the most undernourished might have already died, and thus might have been eliminated from the undernourished sample [86]. Therefore, the relationship between child malnutrition and mortality must be accepted with caution. Nonetheless, the findings are of immense value because they point towards a possible clustering of child malnutrition and mortality in certain geographic regions.

The accentuating negative association between female literacy and mortality on the one hand, and the attenuating positive association between poverty and mortality on the other hand deserve particular attention. The indications are obvious. With the heightened focus and increased investment by the Government of India in maternal and child health programs (Reproductive and Child Health Program, Janani Suraksha Yojana under the National Rural Health Mission) and nutrition programs (ICDS and mid-day meal schemes) and the availability of these programs to those who are below poverty line [87]–[90], there is every likelihood that being poor or rich does not matter given that one has the capacity to access information regarding such programs and that people demand for such services. Under these circumstances the effect of literacy, especially female literacy is likely to increase and the effect of poverty is likely to reduce. However, we cannot completely ignore the effect of poverty on child health. The Mahatma Gandhi National Rural Employment Guarantee Scheme is a welcome step in arresting poverty, especially in the rural areas of the country [91]. It is also worth mentioning that the Government of India is debating on the ‘National Food Security Bill’ which is likely to cover 75 percent of the India's population. The draft ‘National Health Bill 2009’ is also under debate in the Parliament for approval.

### Limitations of the Study

One of the key limitations of the analysis was the non-availability of information on environmental variables such as climate, rainfall, agriculture, etc. Several recent studies have highlighted the importance of such variables in explaining the variations in infant and child mortality [8], [92]–[96]. We could not use such variables in the analysis because of the lack of availability of data on such variables for the different geographic regions of India. However, we could use urban-rural residence in our analysis. Urban-rural residence has been one of most commonly identified spatial factors in mortality analysis. Another advantage of urban-rural residence is that it closely corresponds to population density which is an important factor in spatial patterns of child mortality [8], [97]. Thus, we were able to address some of these factors by using percent urban in our analysis. There is also a possibility of spatial correlations in causes of death over the geographic regions. Though we admit that it is important to address those, we could not do much due to the unavailability of data on causes of death at the regional level. Further studies must utilize information on such variables while explaining spatial variations in infant and child mortality in India.

## Supporting Information

Table S1 **List of regions (based on LISA cluster maps) depicting anomalies in prevalence of infant and under-five mortality by poverty, underweight children, female literacy and level of urbanization in India, 2004.**
(DOC)

Appendix S1 **Details of the technical methods used in the paper.**
(DOC)
