# Supplementary material for: Infant and Child Mortality in India in the Last Two Decades: A Geospatial Analysis
Source: PLoS One. 2011 Nov 2;6(11):e26856. doi: 10.1371/journal.pone.0026856 (PMC3206872; doi:10.1371/journal.pone.0026856)
Supplement: Table S1 — List of regions (based on LISA cluster maps) depicting anomalies in prevalence of infant and under-five mortality by poverty, underweight children, female literacy and level of urbanization in India, 2004. (DOC) [file pone.0026856.s001.doc]

| **Table S1: List of regions (based on LISA cluster maps) depicting anomalies in prevalence of infant and under five mortality by poverty, underweight, female literacy and level of urbanization in India, 2004** | | | | | | | | | |
| --- | --- | --- | --- | --- | --- | --- | --- | --- | --- |
| **Variables** | **Natural Regions** | | | | | | | | |
|  | **High-High** | | | **Low-Low** | | **High-Low** | | **Low-high** | |
| **IMR vs Poverty** | Arawalli Range-Rajasthan | | | North Malabar-Kerala | | Arunachal Pradesh | | South Coast-Andhra Pradesh | |
|  | Banas-Chambal Basin-Rajasthan | | | South Malabar & West Hills-Kerala | | Tripura | | Delhi | |
|  | Malwa Plateau-Madhya Pradesh | | | Central Coast-Kerala | | Mizoram | |  | |
|  | North West Uplands-Madhya Pradesh | | | South Coast-Kerala | | Manipur | |  | |
|  | North East Uplands-Madhya Pradesh | | | Kongunad & Nilgiris-Tamil Nadu | | East valley & Chachar-Assam | |  | |
|  | South West Plain-Uttar Pradesh | | | South East Coast-Tamil Nadu | | South Plateau-Karnataka | |  | |
|  | South Uplands-Uttar Pradesh | | | Mizoram | |  | |  | |
|  | Oudh Plain-Uttar Pradesh | | | Tripura | |  | |  | |
|  | Bhojpur Plain-Uttar Pradesh | | |  | |  | |  | |
|  | South Plain-Bihar | | |  | |  | |  | |
|  | South Plateau-Bihar | | |  | |  | |  | |
|  | Chhattisgarh & Bastar-Madhya Pradesh | | |  | |  | |  | |
|  | Tel-Mahanadi Region-Orissa | | |  | |  | |  | |
|  | South Plateau-Orissa | | |  | |  | |  | |
|  | Coast & Delta-Orissa | | |  | |  | |  | |
|  | North Coast-Andhra Pradesh | | |  | |  | |  | |
| **IMR vs Underweight** | Arawalli Range-Rajasthan | | | North Malabar-Kerala | | South East Coast-Tamil Nadu | | South Coast-Andhra Pradesh | |
|  | Banas-Chambal Basin-Rajasthan | | | South Malabar & West Hills-Kerala | | Kongunad & Nilgiri-Tamil Nadu | | North Coast-Andhra Pradesh | |
|  | Malwa Plateau-Madhya Pradesh | | | Central Coast-Kerala | |  | | Coast & Delta-Orissa | |
|  | North West Uplands-Madhya Pradesh | | | South Coast-Kerala | |  | |  | |
|  | North East Uplands-Madhya Pradesh | | | South Plateau-Karnataka | |  | |  | |
|  | South West Plain-Uttar Pradesh | | | Arunachal Pradesh | |  | |  | |
|  | South Uplands-Uttar Pradesh | | | Nagaland | |  | |  | |
|  | Oudh Plain-Uttar Pradesh | | | Manipur | |  | |  | |
|  | Bhojpur Plain-Uttar Pradesh | | | Mizoram | |  | |  | |
|  | South Plain-Bihar | | | Tripura | |  | |  | |
|  | South Plateau-Bihar | | |  | |  | |  | |
|  | Chhattisgarh & Bastar-Madhya Pradesh | | |  | |  | |  | |
|  | Tel-Mahanadi Region-Orissa | | |  | |  | |  | |
|  | South Plateau-Orissa | | |  | |  | |  | |
| **Variables** | | **Natural Regions** | | | | | | |  |
|  |  | **High-High** | **Low-Low** | | **High-Low** | | **Low-high** | |  |
| **IMR vs Female literacy** | | South Coast-Andhra Pradesh |  | | North Malabar-Kerala | | Arawalli Range-Rajasthan | |  |
|  | | Coast & Delta-Orissa |  | | South Malabar & West Hills-Kerala | | Banas-Chambal Basin-Rajasthan | |  |
|  | |  |  | | Central Coast-Kerala | | Delhi | |  |
|  | |  |  | | South Coast-Kerala | | Malwa Plateau-Madhya Pradesh | |  |
|  | |  |  | | Kongunad & Nilgiri-Tamil Nadu | | North West Uplands-Madhya Pradesh | |  |
|  | |  |  | | South East Coast-Tamil Nadu | | North East Uplands-Madhya Pradesh | |  |
|  | |  |  | | South Plateau-Karnataka | | South West Plain-Uttar Pradesh | |  |
|  | |  |  | | East valley & Chachar-Assam | | South Uplands-Uttar Pradesh | |  |
|  | |  |  | | Nagaland | | Oudh Plain-Uttar Pradesh | |  |
|  | |  |  | | Manipur | | Bhojpur Plain-Uttar Pradesh | |  |
|  | |  |  | | Mizoram | | South Plain-Bihar | |  |
|  | |  |  | | Tripura | | South Plateau-Bihar | |  |
|  | |  |  | |  | | Chhattisgarh & Bastar-Madhya Pradesh | |  |
|  | |  |  | |  | | Tel-Mahanadi Region-Orissa | |  |
|  | |  |  | |  | | South Plateau-Orissa | |  |
| **IMR vs Urbanisation** | | Delhi | South Malabar & West Hills-Kerala | | North Malabar-Kerala | | Arawalli Range-Rajasthan | |  |
|  | | Malwa Plateau-Madhya Pradesh | Arunachal Pradesh | | Central Coast-Kerala | | Banas-Chambal Basin-Rajasthan | |  |
|  | |  | Nagaland | | South Coast-Kerala | | North West Uplands-Madhya Pradesh | |  |
|  | |  | East valley & Chachar-Assam | | South Plateau-Karnataka | | North East Uplands-Madhya Pradesh | |  |
|  | |  | Tripura | | Manipur | | South West Plain-Uttar Pradesh | |  |
|  | |  |  | | Mizoram | | South Uplands-Uttar Pradesh | |  |
|  | |  |  | |  | | Oudh Plain-Uttar Pradesh | |  |
|  | |  |  | |  | | Bhojpur Plain-Uttar Pradesh | |  |
|  | |  |  | |  | | South Plain-Bihar | |  |
|  | |  |  | |  | | South Plateau-Bihar | |  |
|  | |  |  | |  | | Chhattisgarh & Bastar-Madhya Pradesh | |  |
|  | |  |  | |  | | Tel-Mahanadi Region-Orissa | |  |
|  | |  |  | |  | | South Plateau-Orissa | |  |
|  | |  |  | |  | | Coast & Delta-Orissa | |  |
|  | |  |  | |  | | North Coast-Andhra Pradesh | |  |
|  | |  |  | |  | | South Coast-Andhra Pradesh | |  |

| **Variables** | **Natural Regions** | | | |
| --- | --- | --- | --- | --- |
|  | **High-High** | **Low-Low** | **High-Low** | **Low-high** |
| **U5MR vs Poverty** | Arawalli Range-Rajasthan | North Malabar-Kerala | North West Plateau-Karnataka | South Coast-Andhra Pradesh |
|  | Banas-Chambal Basin-Rajasthan | South Malabar & West Hills-Kerala | South Plateau-Karnataka | Delhi |
|  | Malwa Plateau-Madhya Pradesh | Central Coast-Kerala | East valley & Chachar-Assam |  |
|  | North West Uplands-Madhya Pradesh | South Coast-Kerala | Manipur |  |
|  | North East Uplands-Madhya Pradesh | Kongunad & Nilgiris-Tamil Nadu |  |  |
|  | South West Plain-Uttar Pradesh | South East Coast-Tamil Nadu |  |  |
|  | South Uplands-Uttar Pradesh | Mizoram |  |  |
|  | Oudh Plain-Uttar Pradesh | Tripura |  |  |
|  | Bhojpur Plain-Uttar Pradesh |  |  |  |
|  | South Plain-Bihar |  |  |  |
|  | South Plateau-Bihar |  |  |  |
|  | Chhattisgarh & Bastar-Madhya Pradesh |  |  |  |
|  | Tel-Mahanadi Region-Orissa |  |  |  |
|  | Coast & Delta-Orissa |  |  |  |
| **U5MR vs Underweight** | Arawalli Range-Rajasthan | North Malabar-Kerala | South East Coast-Tamil Nadu | South Coast-Andhra Pradesh |
|  | Banas-Chambal Basin-Rajasthan | South Malabar & West Hills-Kerala | Kongunad & Nilgiri-Tamil Nadu |  |
|  | Malwa Plateau-Madhya Pradesh | Central Coast-Kerala |  |  |
|  | North West Uplands-Madhya Pradesh | South Coast-Kerala |  |  |
|  | North East Uplands-Madhya Pradesh | North West Plateau-Karnataka |  |  |
|  | South West Plain-Uttar Pradesh | Malenad & Coast-Karnataka |  |  |
|  | South Uplands-Uttar Pradesh | South Plateau-Karnataka |  |  |
|  | Oudh Plain-Uttar Pradesh | East valley & Chachar-Assam |  |  |
|  | Bhojpur Plain-Uttar Pradesh | Manipur |  |  |
|  | South Plain-Bihar | Mizoram |  |  |
|  | South Plateau-Bihar | Tripura |  |  |
|  | Chhattisgarh & Bastar-Madhya Pradesh |  |  |  |
|  | Tel-Mahanadi Region-Orissa |  |  |  |

| **Variables** | **Natural Regions** | | | |
| --- | --- | --- | --- | --- |
|  | **High-High** | **Low-Low** | **High-Low** | **Low-high** |
| **U5MR vs Female literacy** | South Coast-Andhra Pradesh |  | North Malabar-Kerala | Arawalli Range-Rajasthan |
|  | Coast & Delta-Orissa |  | South Malabar & West Hills-Kerala | Banas-Chambal Basin-Rajasthan |
|  |  |  | Central Coast-Kerala | Malwa Plateau-Madhya Pradesh |
|  |  |  | South Coast-Kerala | North West Uplands-Madhya Pradesh |
|  |  |  | Kongunad & Nilgiris-Tamil Nadu | North East Uplands-Madhya Pradesh |
|  |  |  | South East Coast-Tamil Nadu | South West Plain-Uttar Pradesh |
|  |  |  | Malenad & Coast-Karnataka | South Uplands-Uttar Pradesh |
|  |  |  | South Plateau-Karnataka | Oudh Plain-Uttar Pradesh |
|  |  |  | East valley & Chachar-Assam | Bhojpur Plain-Uttar Pradesh |
|  |  |  | Nagaland | South Plain-Bihar |
|  |  |  | Manipur | South Plateau-Bihar |
|  |  |  | Mizoram | Chhattisgarh & Bastar-Madhya Pradesh |
|  |  |  | Tripura | Tel-Mahanadi Region-Orissa |
| **U5MR vs Urbanisation** | Delhi | South Malabar & West Hills-Kerala | North Malabar-Kerala | Arawalli Range-Rajasthan |
|  | Malwa Plateau-Madhya Pradesh | East valley & Chachar-Assam | Central Coast-Kerala | Banas-Chambal Basin-Rajasthan |
|  |  | Nagaland | South Coast-Kerala | North West Uplands-Madhya Pradesh |
|  |  |  | South Plateau-Karnataka | North East Uplands-Madhya Pradesh |
|  |  |  | Kongunad & Nilgiris-Tamil Nadu | South West Plain-Uttar Pradesh |
|  |  |  | South East Coast-Tamil Nadu | South Uplands-Uttar Pradesh |
|  |  |  | Manipur | Oudh Plain-Uttar Pradesh |
|  |  |  | Mizoram | Bhojpur Plain-Uttar Pradesh |
|  |  |  | Goa | South Plain-Bihar |
|  |  |  |  | South Plateau-Bihar |
|  |  |  |  | Chhattisgarh & Bastar-Madhya Pradesh |
|  |  |  |  | Tel-Mahanadi Region-Orissa |
|  |  |  |  | Coast & Delta-Orissa |
|  |  |  |  | South Coast-Andhra Pradesh |
