# Supplementary material for: Infant and Child Mortality in India in the Last Two Decades: A Geospatial Analysis
Source: PLoS One. 2011 Nov 2;6(11):e26856. doi: 10.1371/journal.pone.0026856 (PMC3206872; doi:10.1371/journal.pone.0026856)
Supplement: Appendix S1 — Details of the technical methods used in the paper. (DOC) [file pone.0026856.s002.doc]

**Appendix S1**

Moran- *I* is the Pearson coefficient measure of spatial autocorrelation. Spatial autocorrelation measures the degree to which data points are similar or dissimilar to their spatial neighbors. Moran-*I* is given by

$$Moran's I=C X \frac{\sum_{ij} w_{ij}z_{i}z_{j}}{\sum_{i} z_{i}^{2}}$$

Z_i_: standardized variable of interest

W_ij_: weight matrix

Negative (positive) values indicate negative (positive) spatial autocorrelation. Positive autocorrelation indicates that points with similar attribute values are closely distributed in space whereas negative spatial autocorrelation indicates that closely associated points are more dissimilar. Values range from −1 (indicating perfect dispersion) to +1 (perfect correlation). A zero value indicates a random spatial pattern.

Univariate Local Indicators of Spatial Association (LISA) measure the correlation of neighborhood values around a specific spatial location. It determines the extent of spatial non-stationarity and clustering present in the data. It is given by

$$I_{i}= z_{i}\sum_{j} w_{ij}z_{j}$$

Bivariate LISA measures the local correlation between a variable and weighted average of another variable in the neighborhood.

$$I_{i}= n_{i}\sum_{j} w_{ij}z_{j}$$

The LISA functionality in GeoDa offers two important options, cluster maps and significance maps. The cluster map is a special choropleth map showing those locations with a significant local Moran statistic classified by the type of spatial correlation: bright red for high-high associations, bright blue for low-low, light blue for low-high and light red for high-low. The high-high and low-low suggest clustering of similar values, whereas high-low and low-high locations indicate spatial outliers. On the other hand, the significance map is a special choropleth map showing those locations with a significant local Moran statistic in different shades of green depending on the significance level. The significance levels are shown as p<0.05, p<0.01, p<0.001, p<0.0001.

Spatial regression models evaluate clustering of an outcome variable not explained by independent variables. Spatial clustering is accounted with reference to the clustering of the error terms. The error term captures the influence of unmeasured independent variables.

$$y= X\beta+ u$$

$$u= \lambda W_{u} + \varepsilon$$

u: model prediction error,


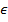
: residues (spatially uncorrelated),

λ: special auto regressive parameter

Under geospatial analyses, we first generated the Moran–*I* statistics for various outcome variables to ascertain the spatial autocorrelation in contiguous natural regions of India across various survey rounds. We then proceeded with univariate and bivariate LISA (cluster and significance) maps to measure the correlation of neighborhood values around a specific spatial location, and to measure the local correlation between a variable and weighted average of another variable in the neighborhood respectively. We then employed aspatial ordinary least square (OLS) models to examine the effect of various socioeconomic and health facility variables on the outcome variables. The final step was to run spatial regression model and generate residual maps to model the spatial clustering of the outcome variable that is not explained by the set of predictor variables and use the residuals in the regression model to account for the spatial clustering effect. All these geospatial analyses were carried out separately for the three survey rounds. Estimates were generated for each of the 76 geographic regions and then analyzed using the above-mentioned geo-spatial techniques in GeoDa software.

To assess the relationship between key exogenous variables with the infant and under-five mortality rates by accounting for both space and time, we use a longitudinal model with a spatial covariance structure that simultaneously adjusts for correlation over space and time. This method treats the space-time correlation as a nuisance by explicitly modeling the correlation structure. This approach is supported by a preliminary analysis of the cross-sectional data using a spatial econometrics approach, which identified spatial correlation as being a nuisance, and, identified the preference for a spatial error model in contrast to a spatial lag model [73]. Similarly, evaluation of residuals indicated that they were normally distributed with homogenous variance. The model takes the following form:

$$y_{it}= \beta_{0}+ I(year)\beta_{1} +X_{it}\beta_{x} + u_{i} +e_{it}$$

where *y_it_* refers to the Infant Mortality Rate (IMR) or Under-Five Mortality Rate (U5MR) in region *i* during year *t*, *I (year)* is an indicator for the year with 2004 as the reference and vector of parameters ***β_1_***, and the bolded ***X****_it_* refers to the vector of independent variables (poverty, underweight, urban, female literacy, and safe delivery) with corresponding parameter vector ***β_x_***. The *u_i_* and *e_it_* are random effects for the region – both assume a Gaussian distribution with mean 0, but the covariance structure for *u_i_* is assumed to follow an exponential spatial structure that takes into account the distance between regions in computing the spatial correlation. We use the AIC and BIC to identify the best model fit and extended this model to test the inclusion of interactions with year. To assess the contribution of a covariate in the interaction models, we used the Type 3 sums of squares, which apply an F-test. Models were run in SAS 9.2-64 bit using PROC MIXED.
